# MASTERS-D Study: A Prospective, Multicenter, Pragmatic, Observational, Data-Monitored Trial of Minimally Invasive Fusion to Treat Degenerative Lumbar Disorders, One-Year Follow-Up

**DOI:** 10.7759/cureus.640

**Published:** 2016-06-13

**Authors:** Jörg Franke, Neil Manson, David Buzek, Arkadiusz Kosmala, Ulrich Hubbe, Wout Rosenberg, Paulo Pereira, Roberto Assietti, Frederic Martens, Khai Lam, Giovanni Barbanti Brodano, Peter Durny, Zvi Lidar, Kai Scheufler, Wolfgang Senker

**Affiliations:** 1 Klinikum Dortmund; 2 Horizon Health Network; 3 Karvinska hornicka nemocnice; 4 Orthopedics, Klinikum Kulmbach; 5 Faculty of Medicine, University of Freiburg, Germany, Neurosurgical Clinic, Medical Center, University of Freiburg, Germany; 6 Orthopedics, Franciscus Ziekenhuis, Rosendaal; 7 Faculty of Medicine of the University of Porto; 8 Centro Hospitalar São João; 9 Neurosurgery, Fatebenefratelli Hospital; 10 Orthopedics, Ons Lieve Vrouw Ziekenhuis; 11 Spine surgery, London Bridge Hospital, London, UK; 12 Istituto Ortopedico Rizzoli, Bologna, Italy; 13 Neurosurgery, Ustredna vojenska nemocnica SNP, Ruzomberok, Slovakia; 14 Neurosurgery, The Tel Aviv Sourasky Medical Center, Tel Aviv, Israel; 15 Neurosurgery, Klinikum Dortmund; 16 Klinikum Amstetten

**Keywords:** minimally invasive lumbar fusion, minimal access spinal technologies, degenerative lumbar disorders, patient-reported outcomes, pragmatic

## Abstract

The objective of the study is to assess effectiveness and safety of minimally invasive lumbar interbody fusion (MILIF) for degenerative lumbar disorders (DLD) in daily surgical practice and follow up with patients for one year after surgery.

A prospective, multicenter, pragmatic, monitored, international outcome study in patients with DLD causing back/leg pain was conducted (19 centers). Two hundred fifty-two patients received standard of care available in the centers. Patients were included if they were aged >18 years, required one- or two-level lumbar fusion for DLD, and met the criteria for approved device indications. Primary endpoints: time to first ambulation (TFA) and time to surgery recovery (TSR). Secondary endpoints: patient-reported outcomes (PROs)--back and leg pain (visual analog scale), disability (Oswestry Disability Index (ODI)), health status (EQ-5D), fusion rates, reoperation rates, change in pain medication, rehabilitation, return to work, patient satisfaction, and adverse events (AEs). Experienced surgeons (≥30 surgeries pre-study) treated patients with DLD by one- or two-level MILIF and patients were evaluated for one year (NCT01143324).

At one year, 92% (233/252) of patients remained in the study. Primary outcomes: TFA, 1.3 ±0.5 days and TSR, 3.2 ±2.0 days. Secondary outcomes: Most patients (83.3%) received one level MILIF; one (two-level) MILIF mean surgery duration, 128 (182) min; fluoroscopy time, 115 (154) sec; blood loss, 164 (233) mL; at one year statistically significant (P<.0001) and clinically meaningful changes from baseline were reported in all PROs--reduced back pain (2.9 ±2.5 vs. 6.2 ±2.3 at intake), reduced leg pain (2.2 ±2.6 vs. 5.9 ±2.8), and ODI (22.4% ± 18.6 vs. 45.3% ± 15.3), as well as health-related quality of life (EQ-5D index: 0.71 ±0.28 vs. 0.34 ±0.32). More of the professional workers were working at one year than those prior to surgery (70.3% vs. 55.2%). Three AEs and one serious AE were considered procedure-related; there were no deep site infections or deaths.

This is the first study evaluating MILIF for treatment of DLD in daily clinical practice. Clinically significant improvements were observed in all endpoints. Short-term post-surgery improvements (four weeks) were maintained through one year with minimal complications. Our results suggest that MILIF has good-to-excellent outcomes for the treatment of DLD in a broad patient population under different clinical conditions and healthcare delivery systems.

## Introduction

In the past 25 years, minimally invasive spine surgery (MIS) has emerged as an alternative to traditional open surgery to treat degenerative lumbar diseases (DLD). MIS allows less extensive manipulation of surrounding tissues while accomplishing the same goals and objectives for target tissues [1-2]. Short-term benefits of MIS include reductions in intraoperative blood loss, postoperative pain, approach-related soft tissue damage and duration of hospital stay vs open methods. MIS is also associated with earlier ambulation and an increased proportion of patients returning to work vs. open surgeries [3-5].

Published, long-term MIS studies have been associated with one or more of the following study limitations: low number of patients, surgeries performed at a single site using a single surgical technique, retrospective observational design, variability in reporting and surgeon learning curve [4, 6-13]. While available data suggests decreased morbidity and at least comparable outcomes vs. open surgeries for the immediate and short-term postoperative period [14], there is limited data to support the long-term superiority of MIS over open techniques [3]. 

A multicenter study reflecting the usual surgical practice was required to observe outcomes and effectiveness of the minimally invasive techniques performed by experienced surgeons.

### Purpose of the study

The objectives of the MASTERS-D study were to observe patients undergoing minimally invasive lumbar interbody fusion​ (MILIF) short-term recovery of one year to document and evaluate the clinical and radiological patient outcomes in both short-term duration (post-operative, four weeks) [5] and mid-term duration (one year) after MILIF using posterior lumbar interbody fusion (PLIF) or transforaminal lumbar interbody fusion (TLIF) techniques for the treatment of DLDs in a broad patient population. 

## Materials and methods

The study methodology has been described in a previous publication by the authors in Pereira et al [5].

### Study environment

The study was initiated by a group of experienced surgeons and conducted in 19 centers across 14 countries (Europe, Canada, and the Middle East). To avoid a potential learning curve effect, all participating surgeons were required to have performed ≥30 pre-study minimally invasive, instrumented lumbar interbody fusion procedures for DLD and surgical indications. Surgical indications, protocols, follow-up length, monitoring, and data analysis were decided before the study was initiated. Each surgeon was asked to contribute a maximum of 30 consecutive patients from their clinic at the start of the study, and each clinical center had a research nurse participating for the collection of data. The study group met regularly (quarterly) over the internet to discuss progress, barriers, or problems. The sponsor (Medtronic) of the study participated in the monitoring of the data, but did not participate in the study design or had any input in the analysis of results.

### Study design and patients

MASTERS-D was a prospective, international, observational study with a one-year follow-up and monitored (NCT01143324) patients with DLD causing back/leg pain referred to a surgical practice with experienced surgeons. All patients who met the inclusion criteria and were willing to participate in the study were enrolled consecutively in order to reduce selection bias. All patients received routine standard of care according to hospital protocol to reflect the real life practice circumstances, and all patients signed an informed consent/patient data release form.

Monitoring

Monitoring visits were conducted throughout the study by monitors from Medtronic and independent professionals from clinical research organizations (CROs). This approach aimed to ensure compliance with protocol, adherence to data collection procedures, accuracy of submitted clinical data, and verify proper maintenance of records for the duration of the study.

Ethics Approval

Ethics committee (EC), institutional review board (IRB), human research ethics committee (HREC) requirements varied across centers and countries. The study was conducted in compliance with the latest version of the Declaration of Helsinki, and laws and regulations of the countries in which the study was conducted, including data protection laws and the Clinical Investigation Plan. The EC, IRB, HREC, data protection authority, and Competent Authority approvals received were as follows: 1. Klinikum Amstetten, Ethikommission fur das Bundesland Niederosterreich am Sitz des Amtes der NO Landesregierung, Abteilung Sanitats und Krankenanstaltenrecht, Austria; 2. O.L.V.Aalst, Local Ethisch Comite, OL Vrouwziekenhuis Aalst, Belgium; 3. Horizon Health Network, East Spine Center, Local Research Ethics Board, Canada; 4. Universitatsklinikum Freiburg, Central ethics committee (Universität Freiburg, Germany); 5. Mediterraneo Hospital, Local ethics committee, Notification to local authority, submission to Data Protection Authority; 6. Tel Aviv Sourasky Medical center, Local Ethics committee, Tel Aviv Medical Center, Israel and Hospital director; 7. Istituti Ortopedici Rizzoli, Comitato Etico Scientifico, Istituto Rizzoli di Bologna, Italy; 8. Fatebenefratelli Hospital, Comitato Etico Scientifico, Ospedale Fatebenefratelli, Italy; 9. Medical University of Gdansk, Local independent medical ethic committee for scientific research of Gdansk University, Poland; 10. Hospital S Joao, Local comissao de etica do Hospital S Joao, Portugal and Submission to Data Protection Authority; 11. Ustredna vojenska nemocnica SNP, Eticka Komisia, Ustrednex Vojenskej Nomocnice SNP, Slovakia; 12. Hospital Clinic de Barcelona, Comite de investigacion del hospital Clinic de Barcelona, Spain; 13. Guys & St. Thomas NHS Trust, Local NHS National Ethics Service, UK. The following centers did not require ethics committee approval by a local agency, and investigator statements were collected and confirmed by investigators: 1. Karvinska hornicka nemocnice, Czech Republic; 2. Klinikum Kulmbach, Germany, 3. Universitatsklinikum Magdeburg, Germany; 4. Marienhaus Klinikum, Germany; 5. Franziskus Ziekenhis Roosendaal, Netherlands; 6. Bergman Clinics, Netherlands; 7. London Bridge Hospital, Ethics Committee.

Eligibility Criteria

Eligibility criteria were broad and based on the approved indications for the devices used in this study. Inclusion criteria were: eligible patients aged >18 years were clinically assessed as requiring a single- or double-level instrumented lumbar fusion for the treatment of the degenerative lumbar spine. All patients were offered to participate in the study and planned to undergo the fusion procedure using PLIF or TLIF techniques to receive a CD Horizon Spinal System (Medtronic Sofamor Danek Inc., Memphis, TN, USA) via the MAST approach (Minimal Access Spinal Technologies, Medtronic Sofamor Danek USA, Inc.) in accordance with the device label. Exclusion criteria were: patients who had previously undergone extensive open lumbar spine surgery other than microdiscectomy were excluded from the study, as were patients with indications for a procedure other than DLD.

Surgical Procedures

The definitions applied as per study protocol are presented in Table 1. The muscle sparing, minimally invasive approach could be performed unilaterally or bilaterally for instrumentation and spinal decompression at the surgeon’s discretion. One or two cages were placed in the intervertebral space to maintain or restore disc height. To achieve interbody fusion, bone grafts and/or substitutes were used. The posterior stabilization of the treated spinal segments was performed using the CD Horizon Spinal System [5]. 

**Table 1:** Surgical techniques and instrumentation definitions for posterior lumbar surgery and instrumentation according to the study protocol

Term	Description
Open lumbar procedure	Surgical technique using a midline approach and requiring a partial or complete detachment of the lumbar fascia and paraspinal muscles to address the spinal pathology and placement of instrumentation
Minimally invasive procedure	Muscle-sparing surgical technique using an intermuscle- or transmuscle-splitting approach, minimizing detachment of the lumbar fascia and paraspinal muscles to address the spinal pathology and placement of instrumentation
Mini-open technique	Instrumentation placement using direct vision of target structures via an intermuscle-splitting approach
Percutaneous technique	Instrumentation placement using radiographic or navigation guidance via stab incisions without direct vision of target structures

### Endpoints and study assessments

The primary objectives of the study were to observe and document patient short-term recovery after surgery [5] which included: a) time to first ambulation (TFA) (defined as the number of days after surgery before patients were able to get out of bed and ambulate with or without assistance), and b) time to surgery recovery (TSR) (defined as the number of days after surgery until patients no longer needed intravenous infusion of analgesic drugs, had no surgery-related complications/adverse events (AEs) impeding discharge, and no longer needed nursing care).

Secondary outcomes were defined as patient-reported outcomes (PRO) and radiological outcomes one year after surgery; PROs included back and leg pain intensity measured by visual analog scale (VAS) scores [15] Oswestry disability index (ODI) scores [16], and EuroQoL five dimension (EQ-5D) scores [17]. Back and leg pain were assessed on a 10 cm VAS scale (0 = no pain, 10 = the worst possible pain) preoperatively, during the hospital stay, at surgery recovery day, at day two after surgery, at discharge, and at all follow-up visits. Disability was rated on a 0% to 100% scale preoperatively and postoperatively at all follow-up visits using the ten-item ODI (0% = minimal disability, 100% = maximal disability); ODI was recognized by Mapi Research trust. Health related quality of life was assessed pre-operatively and at all follow-up visits using the five-item EQ-5D questionnaire (if it was part of standard of care at the study center) with three levels for each dimension: no problems, some problems, or extreme problems. EQ-5D index scores were obtained using the UK population value set (http://www.euroqol.org). In addition, patients completed the EQ-VAS to self-rate their overall health state on a 0 to 100 scale (0 = maximal health-related problems, 100 = minimal health-related problems).

Clinical and radiological outcomes included the documentation of AEs and fusion rate assessment at one year after surgery. The fusion rate was assessed via computed tomography (CT) scan or X-rays at those sites where this assessment was part of standard of care. As per protocol for assessment by CT, the criteria for fusion was bony bridging, and when assessed through X-rays the criteria were bony bridging, <4° motion in flexion/extension views, and integrity of implanted devices. Other clinical endpoints assessed were: documentation of rehabilitation and return to work; reoperation rates, defined as the proportion of patients needing a second intervention at the treated level(s) within one year; proportion of patients needing intervention at an adjacent level; patient satisfaction as assessed by the surgeon; and a change in consumption of pain medication over time compared with baseline. Work status was assessed at the preoperative visit, at 4 weeks and 3, 6, and 12 months postoperatively [11].

Data were collected prospectively by the investigators (surgeons) or the study nurses as per standard of care for each center and included patient medical history, preoperative data, assessment of time from surgery to TFA, TSR and time to discharge. The objective of the TSR day assessment is to record the day when the patient could be discharged based on his actual clinical condition. In some cases the effective day of discharge may be prolonged by factors other than the patient's clinical recovery such as social factors, reimbursement issues, and/or hospital protocol, in these cases the discharge day was not recorded. Postoperative follow-up visits by the treating surgeon were performed according to standard hospital routine with the recommended schedule of a visit at 4 weeks, and 3, 6, and 12 months postoperatively.

Adverse Events

AEs and serious AEs (SAEs) were defined as “any untoward medical occurrence in a subject”, and “an AE that led to death, led to serious deterioration in the health of a subject, led to fetal distress, death or congenital abnormality” respectively. All investigators/surgeons classified the AEs in seriousness and its relation to the surgery, MAST approach, and device (unrelated, unlikely, possibly, probably or definitely). For reporting purposes, all AEs were classified into lowest level terms following the medical dictionary for regulatory activities terminology (MedDRA), version 15.1 (V15.1).

### Statistical analysis

For continuous variables, summary statistics were calculated together with the number of missing and non-missing values. For categorical variables, absolute and relative frequencies (based on non-missing values) were provided. The change in each PRO score was calculated for all patients with a valid observation of both the preoperative and four-week score of the PRO in concern. Changes from baseline in PROs at four weeks postoperatively were analyzed (depending on the Shapiro-Wilk test results on normality) using the two-sided *t-*test for paired comparisons and the Wilcoxon signed rank test with a significance level of 0.05. A similar strategy was used to evaluate changes between baseline and other follow-up scores, and results are presented as mean (± standard deviation (SD)). 

## Results

### Center contribution

Contributing surgeons at each center were instructed to contribute 10-30 consecutive degenerative disk disorder (DDD) patients. Once the maximum number of patients had been attained the center participation for new patients was closed, and only follow up activities proceeded for one year.

### Patient disposition and baseline demographics

A total of 252 patients underwent an MIS procedure of which 233 (92%) patients remained at follow-up for one-year post-surgery. Eleven patients (4.4%) were lost to follow-up, four withdrew (1.6%), and four (1.6%) were explanted resulting in a total of 19 patients who were unavailable for follow-up at one-year post-surgery. Characteristics of the total MIS population were as follows: 56.3% of patients were female; mean age was 53.8, ±11.8 years; mean body mass index (BMI) was 27.7, ±4.6 kg/m^2^; mean duration of symptoms resulting in planned surgery was 28.5, ±38.2 months; mean duration of conservative treatment was 20.7, ±34.3 months; and the main indications for surgery were leg pain (52%), back pain (38.9%), and neurogenic claudication (9.1%). Patient demographics are shown in Table 2. Spondylolisthesis (52.8%), stenosis (71.4%), and disc pathology (93.7%) were the most common preoperative degenerative lumbar pathologies. Of the spondylolisthesis cases, 69% were degenerative, 84% were Grade 1, and 16% were Grade 2 according to the Meyerding classification. 

**Table 2:** Patient demographics and baseline characteristics

Total number of patients	252
Gender (% females)	56.3
Mean (±SD) age (y)	53.8 (±11.8)
Mean (±SD) BMI (kg/m^2^)	27.7 (±4.6)
Mean (±SD) duration of symptoms resulting in planned surgery (mo)	28.5 (±38.2)
Mean (±SD) duration of conservative treatment (mo)	20.7 (±34.3)
Pre-existing conditions relevant to study (%)	37.3%
Previous lumbar surgeries (%)	15.1%
At target level:	
Microdiscectomy open surgery	3.2%
Microdiscectomy minimally invasive surgery	9.1%
Decompression (minimally invasive surgery)	3.6%

### Surgical procedures performed

One-level surgery was performed in 83.3% (210/252) patients; of these, the majority of cases (90.5%) were interbody fusion at the L4−L5 or L5−S1 levels. 16.7% (42 patients) underwent two-level surgery, where fusion was most frequently (73.8%) performed at L4–S1.

First level surgical approaches varied substantially. Unilateral mini-open approaches were more commonly performed (61.9%) either without decompression of neural structures (18.3%) or with unilateral (36.5%) or bilateral (7.1%) decompression. Bilateral mini-open approaches (38.1%) were performed without decompression (5.2%) or with unilateral (24.6%) or bilateral (8.3%) decompression.

A minority of surgeons reported the use of a navigation system (i.e., pre-CT scan (3.6%), 3D fluoroscopy (1.6%), image fusion technique (2.4%), and O-arm imaging (5.6%) (Table 3). Most fixation techniques were bilateral percutaneous (48.8%) or bilateral mini-open (39.7%). For the interbody fusion, most surgeries used an autograft 75.0% (189/252), while 15.9% (40/252) used allograft bone bank, and 45.5% of the autograft were used in conjunction with nanocrystalline hydroxyapatite (86/189).

**Table 3:** Surgery details

Mean (±SD) surgery duration (min)	136.8 (±50.4)
One level	127.7 (±43.5)
Two level	182.0 (±58.3)
Mean (±SD) estimated blood loss (mL)	175.8 (±160.1)
One level	163.9 (±139.7)
Two level	233.1 (±229.0)
Blood transfusion, n (%)	1 (0.4)
Mean (±SD) total fluoroscopy time (sec)	122 (±130.7)
One level	115.1 (±123.9)
Two level	154.1 (±156.6)
Navigation system used, n (%)	
Pre-CT	9 (3.6)
3D fluoroscopy	4 (1.6)
Image fusion technique	6 (2.4)
O-arm imaging	14 (5.6)
Prophylactic antibiotics prescribed, n (%)	248 (98.4)

### Outcomes

Primary Outcomes

Across 252 patients, the mean TFA was 1.3 ±0.5 days, the mean TSR was 3.2 ±2 days, and the mean time to discharge was 6.3 days [5].

Secondary Outcomes at One-Year Follow-Up (PRO)

**Figure 1:**
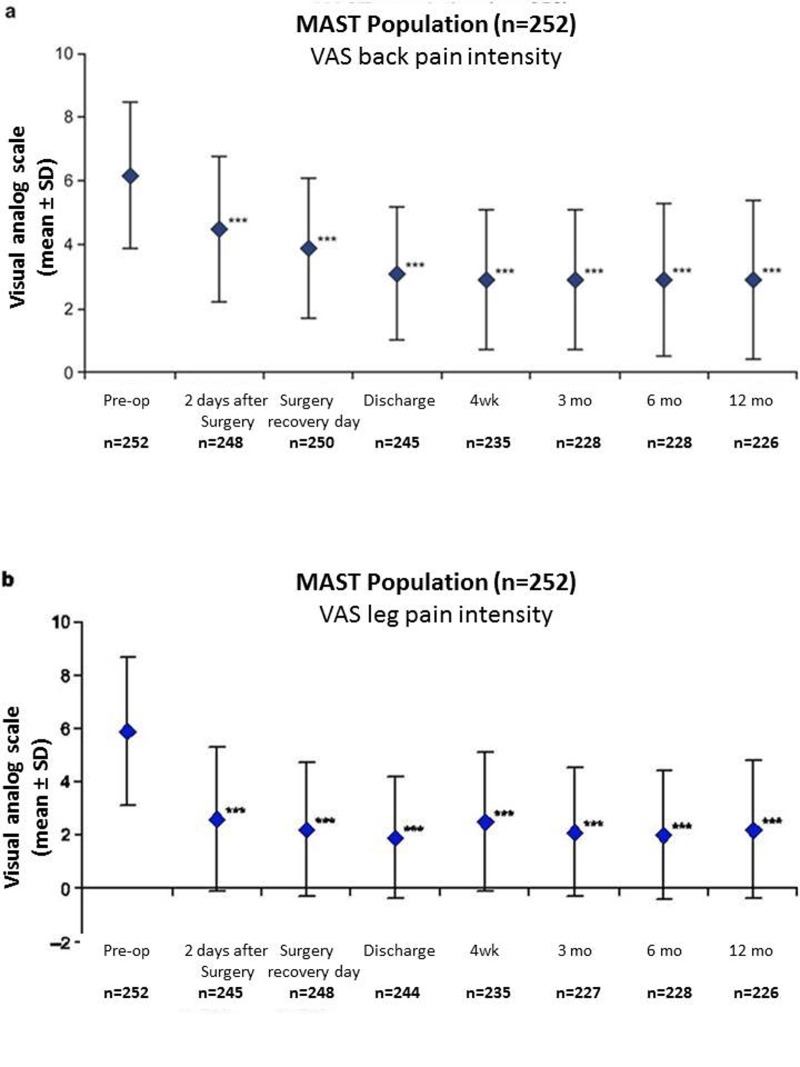
Improvement of back and leg pain. Back (a) and leg (b) pain intensity scores reported preoperatively and postoperatively on a 10 cm visual analog scale (VAS) where 0 = minimal pain intensity or pain frequency and 10 = maximal pain intensity or pain frequency (total population, n=252). ***P < .0001 for difference between preoperative (back 6.2 ±2.3, leg 5.9 ±2.8) and postoperative scores at two days (back 4.5 ±2.3, leg 2.6 ±2.7), surgery recovery day (back 3.9 ±2.2, leg 2.2 ±2.5), discharge (back 3.1 ±2.1, leg 1.9 ±2.3), four weeks (back 2.9 ±2.2, leg 2.5 ±2.6), three months (back 2.9 ±2.2, leg 2.1 ±2.4), six months (back 2.9 ±2.4, leg 2.0 ±2.4), and twelve months (back 2.9 ±2.5, leg 2.2 ±2.6).

**Figure 2:**
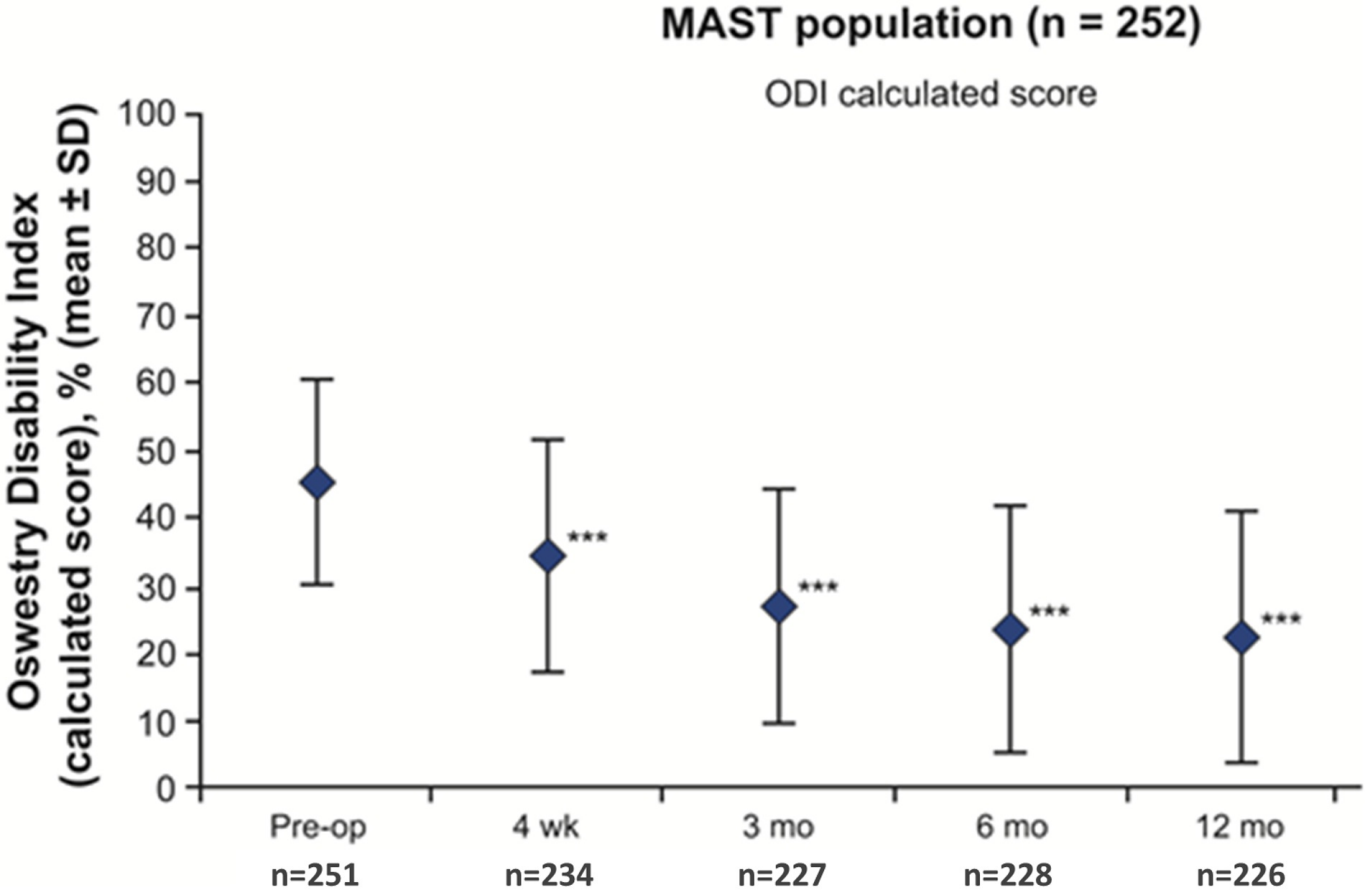
Improvement of patient disability. Oswestry disability index (ODI) scores (calculated in %) reported preoperatively and at four weeks and three, six, and twelve months postoperatively on a 0% to 100% scale, where 0% = minimal disability and 100% = maximal disability (total population, n=252).***P < .0001.

Statistically significant and clinically meaningful changes were reported postoperatively as early as four weeks and were maintained or further improved over one year for each of the PROs assessed. From baseline to one year, there was a 3.3 and 3.8 points improvement in back (2.9 ±2.5 vs. 6.2 ±2.3) and leg pain intensity (2.2 ±2.6 vs. 5.9 ±2.8) respectively, (*P *<.0001; Figure 1). Clinical success in reducing back pain was defined as a minimal change in back pain (≥30% improvement from baseline or a change of ≥1.5 on a 0–10 VAS scale) [18] and was achieved at two days after surgery by 46% and 53% of patients respectively. This increased throughout the study to more than 70% and 72% of patients by one year, respectively. Considering the above-mentioned thresholds for leg pain, clinical success was achieved by 69% and 69% of patients at two days after surgery, increasing to 73% and 74% of patients at one year respectively. Statistically significant reductions in ODI were reported beginning at four weeks and continued through one year, reaching an improvement in score of 22.4% ±18.6 vs. baseline (45.3% ±15.3), (*P *<.0001; Figure 2). Minimally important changes (≥30% improvement from baseline or improvement in ODI score of ≥10) in disability were achieved by more than 41% and 51% of patients respectively at four weeks which increased to more than 70% and 77%, respectively by one year.

**Figure 3:**
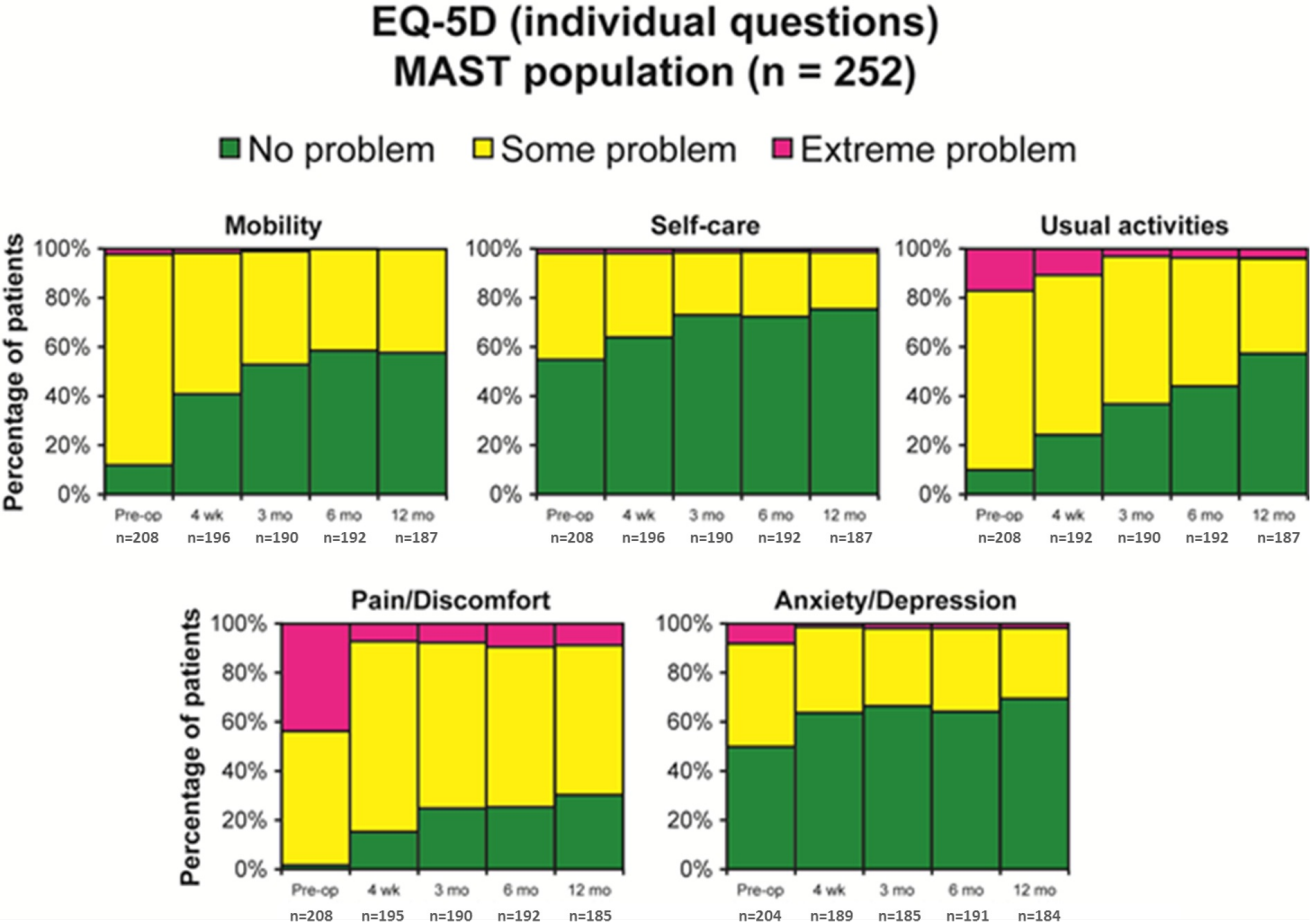
Overall amelioration in quality of life. Percentages of patients who reported on five EQ-5D domains preoperatively vs. four weeks, three months, six months and twelve months.

Improvement in EQ-VAS scores vs. preoperative scores began at four weeks (12.0 points; *P* <.0001) and reached 18.0 points at one year (*P *<.0001). At one year, the proportion of patients who reported no health-related problems increased from the preoperative values in each of the five individual EQ-5D domains: mobility (57.2% vs. 11.1%), self-care (75.4% vs. 54.3%), usual activities (56.7% vs. 9.6%), pain/discomfort (30.3% vs. 1.4%), and anxiety/depression (69.6% vs. 49.5%) (Figure 3). For each domain, improvements were apparent as early as four weeks and continued to increase through one year. The EQ-5D index improved from a mean of 0.34 ±0.32 preoperatively to 0.71 ±0.28 at one-year follow-up.

Clinical and Radiological Surgery Outcomes

Assessment of fusion was not standard of care at all sites, and not all levels treated were assessed for fusion success. One hundred thirty one-level and 24 two-level patients who were assessed for fusion as per protocol at one year; the fusion rates were 90.8% (118/130) and 89.6% (43/48) respectively. Some sites assessed fusion at one year, but used different site-specific criteria than those set out in the protocol of this study; an additional 14 patients (11 one-level and 3 two-level) were assessed using non-protocol defined criteria had fusion rates of 90.9% (10/11 levels) and 100% (6/6 levels) respectively.

The rate of additional lumbar spinal surgeries was low at 2.8% (7/252) in seven patients at six centers. Of these seven additional surgeries, three (1.2%) were reoperations (at the same level--loss of pedicle screw fixation causing recurrent spondylolisthesis and left leg radiculopathy, revision of right pedicle screw S1, vertebral body collapse of upper target vertebra), and four (1.6%) were performed at an adjacent level (Disc herniation on upper adjacent level with extrusion on the right side, pedicle screw dislocation because of osteoporotic bone and trauma, intraspinal hemorrhage at one and two levels above the previously operated levels, and pre-existing lower adjacent level of DDD).

Likely reflective of local clinical practice, fewer than half (45%, 102/226) of the patients were receiving rehabilitation at three months. Assessed by the surgeon at one year, the satisfaction with the results of treatment after the surgery was 81% (177/218), while 78% (171/218) indicated to be helped by the treatment as expected, and 82% (178/218) indicated to have the same treatment again for the same condition. At one year, the surgeons indicated that 85.8% of the patients were completely improved (28%, 61/218), much improved (41%, 89/218), or slightly improved 17% (37/218) with regard to their back pain recovery compared to preoperative levels.

Ability to return to work (full- and part-time work) increased postoperatively among professional workers which composed of 57% of the total patient population (143/252). At the preoperative visit, 55.2% (79/143) were working, while at one year postoperatively, this number climbed to 70.3% (90/128). The rate of professional workers being on paid leave/disability because of back problems was halved from pre-op (57/143, 39.9%) to one year (22/128, 17.2%).

**Figure 4:**
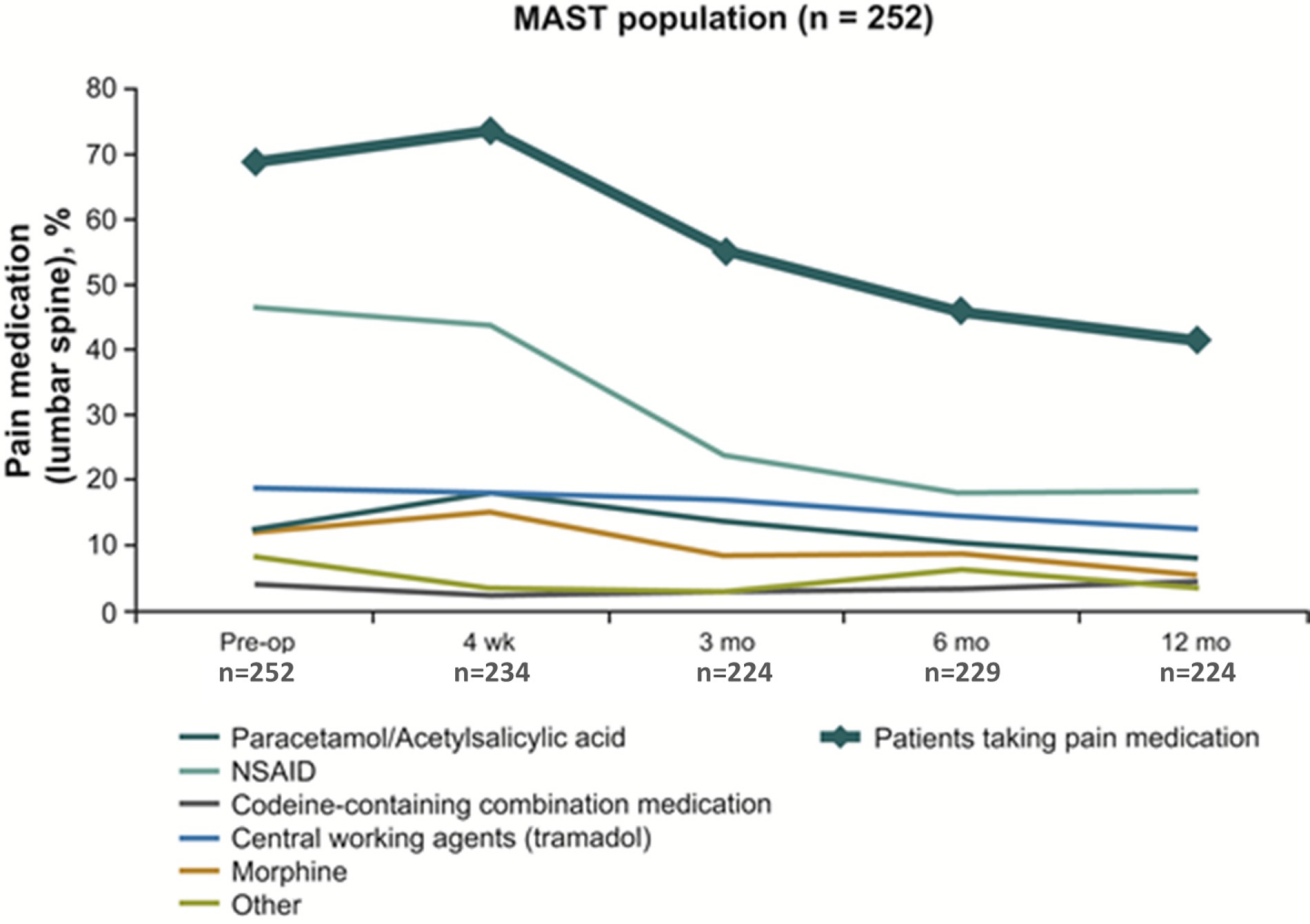
Decreased frequency and potency of pain medication for lumbar spine in the past week. Percentages may not equal 100% because combinations of medications may have been used by the same patient.

Even among patients who were still taking lumbar spine pain medications (68.7% pre-op vs 41.5% at one year), the frequency and potency of pain medication use decreased over the one year after surgery vs. preoperative conditions (Figure 4).

Adverse Events

Throughout the study, 50 AEs in 39 patients were considered by the investigator to be related to surgery, MAST, or device. Nine of these were SAEs (acute allergic reaction, postoperative confusion, leg pain, back pain, lumbar disc herniation, spinal hematoma, and urosepsis). Out of these 50 AEs, 3 AEs (0.79%, 2/252) and 1 SAE (increased leg pain) were considered related to the minimally invasive approach (Table 4); there was one superficial incision site abscess related to surgery (0.4%), and there were no deep wound infections among the 252 patients.

**Table 4:** Adverse events in total population over twelve months * Parentheses indicate a serious adverse event.

MedDRA code low level terms	Count of MAST™ related	Count of total related to device, surgery, or MAST™ (serious event)
Acute allergic reaction	0	1 (1)*
Back pain	0	7(1)*
Confusion postoperative	0	1 (1)*
Dural tear	0	4
Fever	0	2
Hypoesthesia	0	3
Implant site seroma	0	2
Implant site abcess	0	1
Leg pain	3 (1)*	10 (3)*
Lumbar disc herniation	0	1 (1)*
Lumbar radiculopathy	0	3
Nausea	0	4
Sacro-iliac pain	0	4
Spinal hematoma	0	1 (1)*
Urinary tract infection	0	3
Urosepsis	0	1 (1)*
Vertigo	0	1
Vomiting	0	1
Total	3(1)	50 (9)

## Discussion

We reported the one-year results of a pragmatic, multicenter, prospective, monitored study using minimally invasive fusion strategies in the daily surgical practice setting in 19 countries. Our results indicate that MILIF outcomes are in line with other reported surgical studies for the spine and provide valuable insights into the management of DLD in the usual clinical practice. This observational study did not add to the patient burden of illness and has gathered a large network of surgeons who have cooperated to collect a comprehensive set of outcomes including an assessment of long-term outcomes through the achievement of high levels of one-year follow-up (92%).

Short surgery duration and low blood loss achieved with MILIF at four weeks after surgery were beneficial for patients as they aided in faster recovery [5]. These improved clinical results with low complication and reoperation rates were maintained over the one year of the study. Statistically significant and consistent results in all assessed PROs (VAS back and leg pain, ODI, and EQ-5D) were seen from two days after surgery and were maintained throughout the one-year study indicating reduced pain, better mobility, and improvements in quality of life. Clinical success [18] was demonstrated in the rapid and persistent improvement in pain and disability, which started as early as two days after surgery and was maintained or improved throughout the one-year study. Reflective of the improvements in pain and disability, high levels of patient satisfaction with surgery (81%) and reductions in both frequency and potency of pain medications were observed vs. baseline over the one year after surgery. In addition, low levels of formal rehabilitation at one year have been reported (27.0%). Reduction in the use of pain medications after MIS are also consistent with previous reports [11, 14, 19-22]. In a retrospective study, narcotic use decreased from 100% before MIS to 31% by six months postoperatively [21]. Perioperative morphine use was significantly reduced after MIS TLIF vs. open surgery [11] and patients who received MIS had a significantly faster narcotic independence [22].

Patient well-being was indicated by an improvement in their disability and their ability to return to work. At the one-year postoperative time point, the evaluation of work status included both patients who had and had not been working prior to surgery. At one year, 70.3% (90/128) of patients were working compared with 55.2% (43/79) at the preoperative assessment. Moreover, the number of patients on paid leave/disability was halved in one year (39.9% down to 17.2%).  Improvements in the ability to return to work in this study are in agreement with published reports. In a long-term prospective study, more patients were working (>68%) at two to six years after open or laparoscopic surgery for DDD than had worked preoperatively (52%) [19]. Adogwa et al., 2011 [23] reported a faster return to work (8.5 weeks vs. 17.1 weeks) for patients treated with MIS vs. open surgery, while Parker 2012 described 90% of patients who received MIS TLIF returning to work postoperatively vs. 80% of patients treated with open surgery. In another study, 97% of patients who were working prior to MIS TLIF were able to return to work postoperatively [21].

The range of fusion success rate (89.6%–100.0%), regardless of how it was measured, was similar to that of published literature using minimally invasive and open techniques [3-4, 7, 11-12, 20, 22].  The monitoring of our study allowed us to closely follow up the safety of the device and procedures. The majority of surgical procedures in this study was achieved with minimal short- or long-term complications. Throughout the study, 3 out of 50 (1.19%) AEs were considered specifically MIS-related by the surgeons; out of these three AEs, one was a serious event (severe leg pain). The majority of AEs associated with MIS reported in the literature are site infections. We reported a 0.4% infection rate at four weeks and no deep surgical site infections were observed at one-year follow-up. A comparable infection rate of 0.22% was observed in retrospective studies such as a large cohort of patients (N=1274) [24] who received MILIF. In another retrospective study (N=5170), surgical site infections were 4.6% vs. 7.0% (*P *=.037) for two-level MILIF compared with open surgery, and 4.5% vs. 4.8% (*P *=.77) for one-level MILIF vs. open surgery [25], 0.6% vs. 4%, p=0.0005,  n=362 TLIF, n=1133 open TLIF [22].

Reflective of clinical practice, the majority of surgeries performed in this study included unilateral decompression, and a minor number of surgeries were performed with the use of a navigation system. However, the low use of navigation systems may be the cause for higher radiation exposure (122.0 ±130.7) compared to other studies (38.7 s, 71 s, 101 s) [26-28].  In order to minimize the exposure, it is recommended that surgeons use intermittent fluoroscopy and keep an adequate distance from the X-ray tube [28] and navigation systems [29]. It is important to note that the majority of procedures (75%, 189/252) were performed using autografting, often in conjunction with an osteoconductive synthetic bone paste. The most common graft substitute used in combination with autograft was nanocrystal hydroxyapatite (45.5%, 86/189). However, data was only taken from those centers that proactively reported the types of graft substitutes used and may not be reflective of the entire population.

### Limitations of the study

The lack of a direct comparison of outcomes for a controlled group (MIS and open surgery) as seen in randomized controlled trials (RCTs) was a limitation of the study. Our data is reflective of the usual clinical practice of MIS around the world and collected from a non-selected patient population by investigators who were allowed to choose the management strategies normally available to them in their standard practice. This study shows that MILIF is a feasible treatment for DLD, provides valuable insights on how practitioners apply MILIF in their practice, and will facilitate the design of future studies and identify specific indications or patient subpopulations for MILIF. The study fulfills some of the criteria for observational studies [30] such as a large and diverse population of patients in real-world settings, allows for the ability to detect minor treatment effects or rare complications over one year, provides insight into the clinical context in which multiple or complex therapies are delivered and is relatively inexpensive and can be performed rapidly. However, the study does not fulfill the criteria of confounding or selection bias of patients despite the defined inclusion criteria as it is an observational study without a control. It may very well be that the surgeons only selected in their view the 'best' patient for the surgical procedure as in 'real clinical practice'.

### Successes of the study

The study assembled a large group of experienced and willing surgeons to participate in the study. The study was monitored by an independent agency, and the study protocol was decided well in advance. Research nurses were available for data quality and data gathering as little data is missing, and there is a low dropout rate as only 19 patients of 252 dropped out over the course of one year. The short-term results over one year are in line with the literature of other spinal surgeries for DDD. Important data for patients and surgeons was gathered and useful data for the clinical community has been presented. Future studies will have to support or refute the outcomes of this study.

## Conclusions

This is the first pragmatic, international, multicenter, prospective, monitored outcomes study evaluating MILIF to treat DLD under usual surgical practice and to monitor progress over the course of one year. Statistically significant and consistent results were observed for all PROs; patients achieved early mobility, decreased leg and back pain, decreased disability, high patient satisfaction, and more were able to work postoperatively than had been working prior to surgery. This study shows that improvements which were observed four weeks post-surgery were maintained or further improved up to one year with complications. The results suggest that MILIF is an effective, feasible, and safe treatment for DLD in a broad patient population under usual clinical conditions. Further investigation in a larger patient population will provide insights on the long-term benefits of MILIF.
